# Answering the initial 20 questions on COVID-19 (January-February 2020)

**DOI:** 10.7189/jogh.10.010106

**Published:** 2020-06

**Authors:** Igor Rudan

**Affiliations:** Centre for Global Health, Usher Institute, The University of Edinburgh, Scotland, UK

In response to COVID-19 pandemic, the “Journal of Global Health” will publish regular editorials answering the questions related to the pandemic that raised most interest during the preceding period. These editorials will be written in a popular style and aimed at wide audience. They will be based on the best available evidence at the time. This is the first editorial, completed on March 7, 2020, and answering the 20 questions that arose during January and February 2020.

In this text, I have summarised the issues that I see most often lead to misunderstandings or cause confusion about the new COVID-19 pandemic. I’ve offered all of these answers from the perspective of a scientist who has been involved in this field internationally for two decades. My answers seek to give a view that is scientifically based on the data which is currently available about this pandemic, as well as the experience of previous epidemics and pandemics caused by respiratory viruses.

However, I would like to emphasise that there is still a number of ambiguities at the time of writing this article regarding the new COVID-19 pandemic. Scientists are closely monitoring its development and gathering new evidence, which is why this pandemic needs to be taken seriously by all public health experts. In the event of this situation worsening, one should also be prepared for preventive public health measures such as avoiding public gatherings and quarantines at home.

## 1. IS THIS ONE OF “THOSE” INFECTIOUS DISEASES THAT WILL DECIMATE US AND WHICH WILL TAKE ITS PLACE IN HISTORY?

The history of the genus *Homo* and the dozen human species we know of today through archaeological excavation was determined by the fight against infectious diseases. It is likely that the species that are no longer around today are largely extinct due to the spread of infectious diseases [1]. We should be happy that we no longer live in the times of great epidemics and pandemics that once decimated our species. A huge number of people died during their childhood or youth [1].

The most severe mediaeval infections killed up to every third person. Before the discovery of the microscope, people could not even know why it was happening to them. No one thought that tiny, invisible, living microbes could cause these diseases. People assumed that some heavenly punishment for their sins had come upon them. We should, therefore, be grateful that we are living in the age of this advancement of science and medicine [1].

Since 1940, many bacterial diseases have begun to be controlled by antibiotics, and since 1960, many viral and bacterial diseases have had vaccines developed for them [1]. Unfortunately, we do not have a vaccine for all infectious diseases. As we see in the example of this pandemic, new viruses continue to transfer from other species to us because history has not ended with the advent of our generation. Instead, it just continues, but this pandemic certainly does not look like it will “decimate” us.

## 2. COULD SCIENCE HAVE ANTICIPATED THE COVID-19 PANDEMIC?

It could have, because this is already the seventh coronavirus to try to make itself at home within the human population [2]. It is trying to adapt to us and use us as its reservoir. Specifically, we exist together with this virus on a small blue-brown planet in a vast dark universe. We share it with tens of millions of other species. They all strive to barely survive. More than 99%of all species that have ever existed on Earth have failed to survive to this day [1]. That is why viruses constantly transfer themselves to new species and thus expand their reservoirs. They must choose the winning species because they cannot reproduce on their own. Their survival depends on the survival of the species that is their reservoir [3].

Humans are currently an interesting potential viral reservoir. We are expanding rapidly in population size – from about a billion-and-a-half individuals, we have grown to about seven-and-a-half-billion in the last 130 years alone [1]. In doing so, we are conquering new territories, clearing forests, draining wetlands, hunting for pleasure and reducing overall biodiversity [1]. This is making it increasingly difficult for others to survive, while there are more and more of us. Fortunately for us all, the first four human-adapted coronaviruses were merely the cause of common colds [2]. No-one considered the four of them a serious threat to public health.

## 3. WERE SARS AND MERS A SERIOUS THREAT?

SARS (severe acute respiratory syndrome) and MERS (Middle Eastern acute respiratory syndrome) were a real surprise for scientists and experts. These were further respiratory viruses from the coronavirus family, the fifth and sixth that managed to infect humans. Surprisingly, instead of colds, they were able to cause very severe, fatal pneumonia [2]. In addition, the death rate among those infected with both diseases was truly frightening. SARS killed one-in-ten infected while MERS killed one-in-three. Both could have caused a horrible amount of human deaths if they spread to the world's entire population [2].

The coronavirus that caused SARS had its reservoir in bats, which hibernate in caves in winter. This is the time of the year when hardly anyone hunts or consumes bats. However, SARS managed to infect an animal from the cat family, a civet. From a civet it was then also contracted by a man, a farmer in Guangdong province, in late 2002. SARS then spread to more than twenty countries and infected more than 8000 people, with every tenth infected person dying from the disease. It was the fifth human coronavirus, but it was the first to kill humans [2].

Ten years later, in 2012, the MERS coronavirus appeared in Saudi Arabia. It was passed on to humans from the camels in the desert. It has also spread to more than twenty countries, infecting about 2500 people and every third infected person died [2]. So, both SARS and MERS had a really scary case-fatality rate. The advent of MERS showed us that SARS was not an isolated incident with a coronavirus that we could just forget about, but that coronaviruses have become the most significant potential biological threat to our species. If SARS or MERS had spread around the world and infected billions of people, they would have caused a catastrophe unlike any other in the modern history of humans.

## 4. HOW WERE MERS AND SARS DEALT WITH SO SWIFTLY, CONSIDERING THE FACT THAT THEY WERE SO DANGEROUS?

In those two cases, we were actually very lucky. Essentially, an entirely new virus that tries to transfer over to the human species can spread among humans in three basic ways. Each of these three ways informs our strategies for epidemic surveillance and dealing with the epidemic. Once the virus enters the human body, it begins to multiply in the kind of specialised cells that it has the ability to bind to. In the case of coronaviruses, these are the cells of the respiratory system. The virus multiplies in them by “hacking” their cellular genetic code and using their “machinery” to build its own proteins [2].

This multiplication of the virus eventually destroys the infected cells. That is the reason for the eventual development of the symptoms that are characteristic of respiratory infections such as a sore throat and cough. In the first mode of the spread, the virus will “jump” from the infected person to unaffected people only *after* the onset of those symptoms.

Such an epidemic is the easiest to control and contain. That is because the sick person is soon confined to bed and no longer leaves home. Therefore, the virus can mainly spread to their household members. This also makes it relatively easy to identify everyone who was in contact with the person after the onset of symptoms. Those people can then be placed in isolation quite easily. We were fortunate that both SARS and MERS spread to other people only after the onset of the symptoms of the disease. This is why we were able to suppress the epidemics by isolating those infected and all their contacts after the onset of the symptoms. This is quite likely the most important reason why SARS and MERS did not manage to kill remarkably large number of people [2].

It is much more difficult to contain an epidemic if the virus spreads from infected to healthy people during the period of the so-called incubation. This period lasts from the entry of the virus into the body until the onset of the first symptoms [3]. In such case infected persons can transmit the virus by contact to a significantly larger number of people in the days before they get any symptoms. That seems to be the situation with this new COVID-19 pandemic. But even then, it is at least possible for every new patient to determine from whom they contracted the infection. Namely, all newly diagnosed cases had to meet an existing and known case in the past.

This allows epidemiologists to follow the entire chain of movement of the virus from person to person. Due to this favourable circumstance, intensive isolation measures of all those who were in contact with those already infected can significantly slow down the spread of the virus. This was done in Wuhan. That is why “test-trace-isolate”, as the first line of defence, and quarantines as the ultimate response, both represent a justified containment strategy when people are infectious for others during incubation period. Both approaches will slow down and contain the epidemic if applied properly.

A nightmare scenario for any epidemiologist, however, is a third possibility for the virus to spread. In such a variant, people become infected and transmit the virus, but they themselves never show any symptoms. Scientists are currently looking for such possible spreads of contagion with this new COVID-19 pandemic. That is the reason why such possible persons are occasionally mentioned in the media in high-profile articles.

Namely, because of such infected people, cases are beginning to emerge among the population that cannot be linked to any of the already infected people. When people are circulating in the population without symptoms but passing the virus on to others, it is very difficult for epidemiologists to do anything to prevent it from spreading among all humans. Such an epidemic has the potential to spread over time, mainly due to such transmitters or carriers who show no symptoms.

## 5. WHAT MAKES THE NEW COVID-19 OUTBREAK DIFFERENT FROM THE PREVIOUS SIX CORONAVIRUSES?

When something has happened six times within a system that is as complex as the Earth’s ecosystem, then it is no surprise that it would also happen for the seventh time. Another coronavirus is now trying to make itself at home in the human species. In this case, the primary reservoirs were probably bats again. We know this because the genetic sequence of this new coronavirus coincides with that found in hibernating bats in about 96 percent [4]. This time, instead of a civet, a smaller mammal or bird has probably served as coronavirus’ so-called transitional reservoir.

It is possible that it was a pangolin, because in one of them, a coronavirus that matched to the human form by as much as 99% of the sequence was found, although this is not completely definite either [5]. Sequence matching is not the only important factor when it comes to viral “jump” from animals to humans. What is also important is how many individuals in the species that serve as a transitional reservoir are infected and how often the species comes into contact with humans. Sometimes these factors are more important, so they can bridge the gap of 2%-3% of the difference in genome sequence, because pangolins are a protected species and cannot be eaten.

Thus, in the Chinese province of Hubei, in the City of Wuhan, which has a population of eleven million, at around the end of 2019, the number of patients with an unusual and very dangerous type of pneumonia began to increase. Many of them had in common that they visited a particular fish market. It was soon discovered that this infectious disease was spreading very quickly. Each infected person managed to further infect as many as two to three people. Such a degree of infectivity is quite high and leads to rapid growth of the epidemic. We had the misfortune that the disease caused by the 7th coronavirus – COVID-19 – apparently manages to spread even during the incubation period, possibly even by touch.

This incubation period lasts about five days on average. It would be very tricky if it lasted longer, because the novel coronavirus would then have more time to spread from an already infected person to others. However, an incubation period of up to two weeks is not entirely unusual. Even rare cases who had incubation period of up to four weeks seem to be possible. During some of this time, the infected person can spread the virus before the onset of their first symptoms. If there are infected people who do not develop symptoms, it will be really difficult to contain this pandemic until we develop a vaccine. In conclusion, infectivity, ie, the ability to move from the infected to the uninfected, seems to be significantly higher in COVID-19 than it was with SARS and MERS. As a trade-off, we now know that the COVID-19 case-fatality rate is significantly lower than that of SARS and MERS.

## 6. WHAT IS THE CASE-FATALITY RATE AMONG THOSE INFECTED WITH THE COVID-19 EPIDEMIC, AND WHY IS THERE SO MUCH AMBIGUITY ABOUT IT IN THE MEDIA?

In order to answer this rather complicated question, it must first be said that nowadays, the registered number of infected persons and the number of deaths can be monitored online [6]. When the number of deaths was divided by the confirmed and registered number of infected people at the beginning of the pandemic, a figure of about 2% would be obtained [6]. From this alone, one could apparently conclude that – given that this is an entirely new coronavirus and nobody is immune to it – it will necessarily spread throughout the world and infect us all. If it kills 2 percent of all people, then it follows that out of a total of 7.5 billion people about 150 million will necessarily die. It is difficult for anyone who is not a specialist in this field to understand how such an outcome can now be prevented, because a vaccine against this virus does not yet exist, nor do medicines.

The question of the case-fatality rate among those infected with COVID-19 exposed a general lack of epidemiological knowledge among the general public and in the media. From the very beginning of this pandemic, there were people who claimed that the new coronavirus was a disease milder than even the flu, but also those who believed it was significantly more dangerous. In recent days, this issue has finally caught the attention of all the world’s media as the World Health Organisation reported “that about 3.4% of those infected with coronavirus have died” [7]. That sounded terrifying to the media and the public.

But then the President of the USA, Mr Donald Trump, also made a public statement saying the number released by the WHO was “wrong.” He said that “he'd talked to people who knew something about it and that his impression was that the number was certainly below 1%, if not significantly less” [8]. In my guest appearance on Sunday at 2 (an influential Croatian TV programme) on March 1st, I made an estimate of the case-fatality rate of 0.5%-1%, allowing for the possibility that it could even be smaller [9]. Namely, at this point we do not properly distinguish infection-fatality rate from case-fatality rate. So, once that insights into seroprevalence become available, they may even bring this estimate further down.

However, both President Trump and WHO are actually right, each in their own way, which shows best how difficult it is for many people to keep track of what is really going on because of their lack of knowledge of the epidemiology.

In the beginning of every epidemic of a new virus, the virus has to “jump” over from animals to humans, and then from humans to humans. That can be quite difficult. Therefore, the virus will be more likely to successfully infect those with a weakened immune system, who will find it more difficult to reject it. Because of this, the first patients are often people who are either older or already have some underlying illnesses that make them more vulnerable. They end up in a hospital, where at that point no one suspects that pneumonia that they are experiencing may have an epidemic potential.

Then, they infect other hospital patients and some health care professionals. The latter can then spread the disease to other patients in the hospital – mainly to those most susceptible, such as those ailing or immunocompromised, treated for serious illnesses or the elderly. This is the reason why the case-fatality rate among all COVID-19 patients was initially very high in Wuhan and later in Italy. Many people died of COVID-19 infections in hospitals and they were mostly very old and sick people [10].

In the meantime, the virus has started to spread among the general population – in the community and outside of hospitals. It has infected many people who are otherwise healthy and had a much better immune response. A large number of these people would have thought that they had a cold, or the flu, and perhaps even a more severe flu. They were just resting at home and letting those respiratory infections run their course. Considering that it was flu season in Wuhan at the time of the outbreak, and the media reported that a strange epidemic was causing many deaths in Wuhan’s hospitals, it is quite likely that many Wuhan residents who were infected with the coronavirus stayed home and treated themselves.

Few people would choose to go to the hospital to test whether their flu-like symptoms were caused by the novel coronavirus when a deadly epidemic was spreading there. Only the few who have struggled with fevers and symptoms for more than eight or nine days sought help from Wuhan hospitals. In China, there is typically no primary health care and family medicine as we know it, but there are large hospitals in their huge cities where patients report directly. That is the most likely explanation as to why the case-fatality rate among patients at Wuhan hospitals at the beginning of the epidemic was so frighteningly high.

## 7. IS THE INFECTION OF THE MOST VULNERABLE IN WUHAN HOSPITALS EARLY ON IN THE EPIDEMIC THE SOLE REASON FOR THE HIGH INITIAL CASE-FATALITY RATE OF COVID-19?

It is not. The epidemic seemed even more dangerous at first, as it created a great deal of pressure on hospital intensive care units, which were unprepared for this infection. As a result, all severely ill patients could not receive intensive care. This further increased the case-fatality rate at the beginning of the epidemic. That is why the Chinese have started building the two new hospitals. They wanted to ensure a sufficient capacity to provide intensive care. Also, they needed to move all those infected with COVID-19 away from other seriously ill people who were sick from other diseases and at the highest risk of dying if they became infected.

Based on this, it should be understood that the total number of those infected with COVID-19 in Wuhan could have been much higher than what was confirmed by their health statistics. Specifically, only those with coronaviruses who were eventually admitted to the hospital were confirmed to be infected and were tested for the new virus there. They are by no means representative of all those infected with the new coronavirus in Wuhan.

Therefore, the early estimates of case-fatality rates in Wuhan cannot be extrapolated even to all those with confirmed coronavirus infection in Wuhan, let alone would they be representative of the entire population of Wuhan. For those reasons it is wrong to look at the number of deaths and the number of confirmed infected cases and divide those two numbers and draw any conclusions [11].

## 8. WHY DOES THE NUMBER OF INFECTED AND DECEASED PEOPLE ON THE INTERNET, WHICH IS CONSTANTLY BEING UPDATED, GIVE A WRONG IMPRESSION ABOUT THE CASE-FATALITY RATE OF COVID-19?

If all the reported deaths are divided by the total number of people with confirmed infection, then both the numerator and the denominator are wrong when it comes to calculating the actual case-fatality rate. Even deaths in the numerator would be wrong. This is because we monitor the confirmed infected and those who have died in real time. A great many infected people have not even had a chance to either recover or die, so the number of deaths in the numerator is an underestimate.

Some of the people who are presently in intensive care are expected to die in several days, weeks, maybe even months. This is why the number of deaths that corresponds to the number presently infected will inevitably increase over time. Therefore, some future number of deaths, as the numerator, will increasingly correspond to the current number of registered infected persons, as the denominator. As a result, the case-fatality rate of “deaths among those with confirmed infection” will then no longer be 2 or 3 percent, but it may increase quite considerably over time – perhaps to 6-7 percent, or even more.

Therefore, to simply say that “about 3.4% of those confirmed to have been infected so far have died” is not really wrong in itself, which is what the World Health Organisation did. But it missed explaining that this case-fatality rate among confirmed infected people is quite unrepresentative of the mortality rate among all those who were infected [11]. The true rate should be much lower. It would not surprise me, as an epidemiologist, if it is up to ten times smaller, perhaps even more [11]. Depending on the actual, true number of infected people which should be used as a denominator, this could ultimately make COVID-19 a less deadly disease even than the common flu.

## 9. CAN WE BE CERTAIN THAT THE TRUE NUMBER OF INFECTED PEOPLE IS MUCH HIGHER THAN THE NUMBER OF CONFIRMED INFECTED PEOPLE – IS THERE ANY EVIDENCE FOR THIS?

Given that the virus is new and unknown, this is a key question. Unfortunately, the possibility that this virus is quite different from other known viruses must also be allowed. It may be that the case-fatality rate among confirmed infected people is only 3 to 5 times higher than the infection-fatality rate, ie, the number of deaths among all infected people. So, it may not be ten or thirty times higher, as many epidemiologists would expect. The only thing we can be reasonably sure of is that the number of confirmed infected people was not equal to the number of all infected people in Wuhan. The current global totals of confirmed infected and fatalities for COVID-19 are still largely determined by what happened in Wuhan at the beginning of the epidemic. This is because about two-thirds of cases worldwide still originate in Wuhan to this day [6].

That is why I have already explained on “Sunday at 2” TV programme that the case-fatality rate among those who were confirmed to be infected is not so important. It is only a subset of the patients with the most severe symptoms. We need to find out what is the case-fatality rate among all infected people. However, no one can know that at this time, because a random sample of at least 100 000 Wuhan residents would need to be tested for this. Then, the presence of antibodies against coronaviruses should be detected. This is how we measure “seroprevalence” [3].

This would allow us to estimate the number of people in Wuhan who got over the infection with the novel coronavirus without ever seeing a doctor. So far, no one has conducted these studies because the health system was preoccupied with diagnosing and treating the epidemic of COVID-19 in hospitals and struggled with it. It is now understood that during some days of the Wuhan outbreak there were not enough tests for all those who reported to hospitals with symptoms.

However, additional lines of evidence are beginning to emerge. The first is the report of an international panel of experts who visited China [12]. They looked at the cases across China that were reported after February the 1st. At this point, the identification of those infected was significantly improved. Also, hospitals were better prepared for the epidemic. Case-fatality rate of all cases that came under health surveillance and were tested dropped to about 0.7% [12].

For anyone who may remain skeptical of China’s data, the first reports have recently been provided by South Korean authorities. That country has really led by example in proactively testing people, seeking out all those infected and their contacts, isolating them and treating them. In their analyses to date, case-fatality rate of all infected people has been slightly above 0.6% [13]. Both of those estimates could still increase, albeit not substantially, if they also included those who will likely die over time and are currently counted as infected. Still, at such a low case-fatality rate there should not be many additional deaths. In fact, it is more likely that many infected people remained undiagnosed and that the case-fatality rate is actually even lower [11].

Another interesting recent new source is the study of more than 1000 hospitalized COVID-19 patients who were followed up until the very end of the infection. This sample has been collected from more than 500 Chinese hospitals and the result was published recently in a leading medical journal [14]. This analysis quoted their overall case-fatality rate to be about 1.4%. However, it did not include all those infected again, but rather those who requested hospital treatment [14]. Therefore, it should still be possible that the infection-fatality rate is at least two to three times smaller among all those who were infected with the novel coronavirus.

Thus, it appears that data from very different and increasingly reliable sources are starting to converge to the values which I predicted on “Sunday at 2” TV show on March 1st, ie, 0.5%-1%. Because of all of the above, President of the USA Donald Trump was most likely right to say that this number should be closer to 1% and that he believes that it could even be well below 1% [8]. Everything we know about epidemiology and previous pandemics gives us hope that this should be the case.

## 10. CAN EVERYONE IN ANY GIVEN COUNTRY BECOME INFECTED WITH A NOVEL CORONAVIRUS? IF SO, WOULD THE CASE-FATALITY RATE OF 0.5%-1% BE APPLICABLE TO THE WHOLE POPULATION?

The virus will not succeed in infecting everyone for a number of reasons. Our first line of defence consists of anti-epidemic measures. All those who are experiencing symptoms and may be infected are being tested. All their contacts are also tested for the presence of the virus. Then, COVID-19 patients and all their contacts are being isolated [15].

These measures will significantly slow down the spread of the infectious disease and buy us time. It is of utmost importance that the number of patients with COVID-19 does not increase too quickly. When the new cases emerge more slowly, the staff of national health care systems will be able to provide quality care to all patients. For the more severe cases they will also offer intensive care. In the absence of these measures, there would be an exponential increase in the number of infected people. This number would soon outgrow the capacity of the health system. Many people within countries are also protected by their geographical dispersion, ie, many people live in smaller towns and settlements. A large number of them will probably never be exposed to an infected person.

Furthermore, as people become infected, develop COVID-19 and then get well, they should become immune to the virus. As a result, there will be fewer people the virus can still infect. At some point, the number of susceptible persons that infected people can spread coronavirus to will decrease substantially. At some point each infected person will, on average, pass the virus to less than one susceptible person. In time, this will eventually limit and stop the epidemic. That is the reason we vaccinate. Even if the virus infects some unvaccinated people, it will have very few options for further spread. Vaccinated people will already be immune to the virus and their bodies will react vigorously if the virus tries to enter [16]. Many processes in nature are self-limiting in a similar way – forest fires, stock market crashes and epidemics.

Furthermore, the case-fatality rate should not be directly applicable to the entire population of a country to estimate the possible death toll. The first reason is that the virus mainly endangers the elderly, whose case-fatality rates are much higher. Young people and children rarely get unwell and their death rates are much, much lower. This is why age and sex structure of the population can diminish the potential of the virus to cause a very high number of COVID-19 casualties if the population is reasonably young [11].

## 11. SHOULD WE THEN FEAR THE COVID-19 PANDEMIC, AND IF SO, HOW MUCH?

The situation should be taken seriously and people should be cautious, but there is no reason to be overly afraid. There is especially no reason to panic. Many people are afraid of this pandemic because they probably think that we are in a completely unfamiliar situation, so anything could happen. But it is unlikely that much could happen for which science could not find explanations and answers and the epidemiological services could not respond in a timely manner. It is unwise for a serious scientist to try to predict the spread of a completely new and unknown virus to the entire human population in the world, let alone to predict more specific outcomes. Still, we have in recent weeks collected enough information about the new COVID-19 virus for at least some predictions.

If the new coronavirus spreads across any country over time and manages to circumvent the many prevention measures in place, the application of anti-epidemic measures should still limit its casualties to make them at least roughly comparable to the deaths from flu or road accidents in the same period. This means that some healthy caution is advisable. This caution is reasonable as long as it is on the same level as the fear you may feel when sitting in a car and preparing for a longer trip, or when you hear on the news that a more severe form of influenza has arrived.

But many are wondering why the novel coronavirus attracts such a level of media attention. This is because flu has been a well-known disease for decades. It comes back every year and we have experience with its manifestations among many millions of patients worldwide. We know how to develop vaccines against flu in advance of its season [16]. We have even started to produce the first somewhat effective drugs and offer them on the market [17]. Unlike the flu, the new coronavirus is unknown to us. We should remain cautious so that we do not get unpleasantly surprised. At the same time, the most vulnerable among us, who are already seriously ill or very old, are not vaccinated, as is the case with the flu. This is why the new disease, COVID-19, can kill more easily.

## 12. IS IT CLEAR THAT COVID-19 IS A SIGNIFICANTLY MORE DANGEROUS ILLNESS THAN THE FLU?

This question has constantly been being raised since the beginning because many are looking at various figures without a deeper understanding of their background and are comparing the incomparable.

First of all, the general public underestimates how serious and dangerous and serious the flu actually is – especially for the most vulnerable, the elderly and those who are already ill. Globally, influenza causes between 250 000 and 650 000 deaths annually, depending on the strain of the circulating virus [18]. Different strains can cause milder or more severe symptoms, and the virus mutates each year. However, we try to protect those most vulnerable before the flu season begins by vaccinating them.

Therefore, the number of deaths from influenza is reduced by preventive health intervention, ie, vaccination. This cannot be done with the spread of COVID-19. This is the first reason why the flu seems less dangerous than COVID-19. However, it may not be much less dangerous inherently, especially during seasons when flu is more severe. It is just that we protect the most vulnerable. In addition, flu vaccinations make it more difficult for it to spread among the population because there are fewer options for it to transfer to the uninfected. Due to the slower spread, new cases emerge more slowly and the health care system has time to deal with them properly, especially if they require intensive care.

Another reason is that the number of deaths directly from the flu is several times lower than the number of deaths that are indirectly caused by the flu. Influenza is often not cited as a direct cause in statistics on the causes of death, if it has merely led to the exacerbation of some of the long-present chronic, underlying disease. These chronic diseases are then cited as the primary cause of death, rather than influenza. Therefore, the actual role of influenza in annual mortality is often significantly underestimated. It can be several times higher when the causes of death are reclassified at the end of each year, based on the increase in deaths from chronic diseases during the flu season.

The third reason for caution in comparing COVID-19 and flu is that we have a much better idea of the total number of people truly infected with the flu than we do about the novel coronavirus. This is because influenza is a disease that is typically managed within primary care, after which patients are referred to home care. Only the most serious cases of flu are referred to hospital.

There is an obligation to report the total number of people infected with influenza in the population to the central registry, as well as for sick leave. This makes the denominator for case-fatality rate of influenza better known to us than we can estimate it for the novel coronavirus. COVID-19 has so far been diagnosed and treated exclusively in hospitals, where severe cases clustered. The early estimates of case-fatality rates for COVID-19 included patients suffering from hospital outbreaks, which affected the elderly, the sick and the immunocompromised. This is why those case-fatality rates are hardly comparable to a spread that would occur in the general population.

From all of this, it should be concluded that case-fatality rates from influenza, as another very dangerous viral disease, have been mathematically reduced compared to the current reports of case-fatality rates for coronaviruses for the three reasons I mentioned. The first is because we vaccinate those most vulnerable to the flu. The second is that health statistics do not assign the majority of flu deaths to influenza, but rather to the exacerbations of pre-existing underlying illnesses such as cardiovascular diseases, diabetes, malignant tumours and others. The third is that the denominator we use to calculate case-fatality rates from influenza is much closer to the actual number of those infected, while the denominator for coronavirus is not yet known with sufficient certainty. From their experience with other respiratory viruses, epidemiologists know that all events in hospitals will dramatically over-represent the most vulnerable. This is why those case-fatality rates should by no means be applied to the general population.

However, lay members of the public cannot have a good sense of these nuances. That is why it is best to avoid comparisons between case-fatality rates for the flu and for the novel coronavirus until we learn more on the seroprevalence of the latter. The general public underestimates the risk of influenza for the three reasons mentioned above. They are also likely to overestimate the risk of the new coronavirus due to its intense media focus. If influenza infections and deaths were monitored in the same way each year, the public would realise how dangerous the flu really is and how reasonable it is to be vaccinated against it.

Therefore, it still does not seem possible to me, at least at this stage of the pandemic, to decisively state which of the two diseases is inherently more dangerous to human beings or which will eventually cause more human deaths in 2020. Flu will cause fewer deaths because the most vulnerable will be vaccinated and health statistics will not properly attribute many indirect deaths to flu which really caused them. COVID-19, in turn, will cause fewer deaths due to epidemiological surveillance, the prevention of its spread and possible strict quarantine. It may also have seasonal characteristics and simply disappear with the arrival of late spring.

## 13. ARE SUCH STRICT AND MASSIVE QUARANTINES JUSTIFIED?

When we have no other means of defending ourselves against the new virus, all we can really do is retreat indoors and prevent the virus from “jumping” from infected to healthy individuals too quickly. People generally do not have an intuitive sense of exponential growth. If each newly infected person infects just one more person each day, the number of newly infected people will increase from 2 to 16 during the early phase, which does not seem like a big increase.

A little later, it will jump to 1024 infected people from 128 over the next three days, and that does not sound so terrible either. But there will also come four days in which the number of newly infected people will increase from 100 000 to 800 000.

When China realised that the COVID-19 epidemic was out of control and that it already entered this explosive phase, the authorities immediately cut off Wuhan and then fifteen other cities from the rest of the country. In addition, the authorities ordered that the population within these cut-off areas stay in their apartments and not leave. It was an unprecedented measure in human history – tens of millions of people were quarantined for weeks. Everything stopped [12].

However, this measure produced excellent results and China could keep its death toll below 5000, although the epidemic caught them unprepared and the virus spread to all Chinese provinces. A recent report from the World Health Organisation’s commission made up of 25 international experts visiting China concluded the following:

“China’s bold approach to contain the rapid spread of this new respiratory pathogen has changed the course of a rapidly escalating and deadly epidemic”. Faced with an unknown virus, “...China has rolled out perhaps the most ambitious, agile, and aggressive disease containment effort in history”. [12]

## 14. WHAT HAPPENED ON THE DIAMOND PRINCESS SHIP, WHICH IS ALSO ISOLATED? IT APPEARS TO HAVE A CASE-FATALITY RATE OF MORE THAN 1% FOR THOSE INFECTED. ISN'T THAT VERY INFORMATIVE FOR SCIENTISTS?

This may be because, on these large ships, people are mostly older. One should look very closely at the age and gender structure of passengers, which would tell us more about the final outcome. It is also quite possible that a mutated version of the virus, which is somewhat more dangerous, could have entered such a “pocket”. Such isolated groups can always emerge and the disease may actually run a more severe course there. This is possible, but not very likely. Also, this case-fatality rate should not be extrapolated to the entire country for the same reason.

Once the virus begins to spread through the entire human species, it will continue to mutate. It will try to adapt to humans as quickly as possible. According to previous epidemiological experiences, many of those mutations should make it less dangerous for human health, as this would lead to a better adaptation. However, some random mutations could make it more dangerous to us. This is why we need to be on our guard until we get better acquainted with the virus and the pandemic is over. It is unlikely that the novel coronavirus will mutate to become significantly more dangerous than it is now, but we will only be able to assert that with certainty when the pandemic is over.

## 15. WHAT IS HAPPENING IN ITALY AND IRAN? DOES COVID-19 BEHAVE DIFFERENTLY IN THOSE TWO COUNTRIES THAN IT DOES IN OTHERS? COULD THE DEVELOPMENT OF A MUTATED, MORE DANGEROUS VARIANT OF THE VIRUS EXPLAIN THE DIFFERENCES?

These are very difficult questions to answer until we get more quality data from both countries. In principle, it is possible that the first entry of the virus into a new country may be through an infected person in whom the virus has mutated into a more severe form. If all further cases arise from that mutant, then the situation in those countries may initially seem more difficult than elsewhere. In population genetics this development is known as the so-called “founder effect” [19]. However, patterns of spread and case-fatality rates in both countries may also have many different explanations.

The reason why the death toll among infected people in Italy appears to be very high is because the disease is spreading in small-town hospitals and elderly care homes which were completely unprepared for the epidemic. The elderly and infirm are at much greater risk of dying if they become infected.

Case-fatality rates in hospital outbreaks where older, sick and immunocompromised people are affected will be much higher than those in the community, among younger and healthy people. Among the first several hundred deaths in Italy, almost all people were over 60 years of age and had underlying illnesses [20]. That is why the case-fatality rate seems so high there, but it is not representative of the entire population. A harsh flu season would probably have done similar damage if people had not been vaccinated against it. But it is also possible that there are many more cases in the population than previously thought because the virus has been spreading for a long time. In Iran, however, the situation is unclear so far. The most likely explanation, too, is that there are already significantly more cases of infection among the population than it was thought initially.

## 16. IS IT POSSIBLE THAT CORONAVIRUS MAY SURPRISE US AND ULTIMATELY PROVE SIGNIFICANTLY MORE DANGEROUS THAN SEASONAL INFLUENZA? COULD IT PERHAPS CAUSE MORE THAN ONE MILLION DEATHS IN THE WORLD, OR EVEN SEVERAL MILLION?

If COVID-19 proves to be significantly more dangerous than seasonal influenza, then one million deaths worldwide could indeed be expected, perhaps significantly more. Unfortunately, such a scenario is still possible in principle with a virus that is new and unknown to us, for a variety of reasons. Because of this, all experts in the field, including myself, are constantly urging people to take caution, but without the unnecessary panic.

In which scenarios could the situation become much more difficult? First, most epidemiologists, based on their experience with previous epidemics and pandemics, expect the COVID-19 case-fatality rate to fall below 1% when the total death toll is divided by a better estimate of the total number of infected people. However, the virus is new, so it is possible in principle for this specific virus that the number of infected individuals who are not confirmed through testing may not be as large as epidemiologists would expect. This would come as a surprise to science and would indicate a different nature of this virus.

The new coronavirus causing COVID-19 is somewhat similar to that caused by SARS. The SARS pathogen, however, had a much higher case-fatality rate. If the total population infected by the novel coronavirus is found to be higher than the registered infected population by only two or three times, and not by eg, ten times or more, then the case-fatality rate of those infected with COVID-19 could be significantly higher than the flu.

Combined with the lack of an available vaccine, in such a case it would lead to a significantly higher number of deaths than the flu. However, such an occurrence should still be prevented by measures of isolation of the patients and their contacts, as well as by quarantines, which are not applied in the case of flu. We also hope that with the winter ending and the new seasons arriving, seasonality would slow down or completely hinder the further spread of the virus.

Furthermore, the virus could spread to infected people in some of its more dangerous forms, as well as in milder forms. Previous experiences with epidemics have shown that mutation into milder forms is more likely, but mutation into more dangerous forms, or those that are more easily spread, is not impossible either [3]. In some countries, such a variant would increase the case-fatality rate locally compared to other countries or accelerate infection.

This would put their health systems in a really difficult situation as intensive care units would soon become overburdened. With poorer care available, case-fatality rate of all those infected would increase further, with the likely collapse of part of the health system. It is also a very tricky scenario in which many health care professionals would become infected over time in providing care to patients, which would make the situation worse.

Therefore, currently, perhaps the most important citizens of any country are health care professionals who work in hospitals for infectious diseases, especially in their intensive care units. They should be protected not only from work overload, but also from coronavirus infection by their patients. With the increasing number of infected people with severe forms of disease, the demand for quality intensive care, respirators and ECMO devices for extracorporeal oxygen enrichment will become the “bottleneck” of the health care system. The same is true for healthy and well rested doctors and nurses in these wards. Those parts of the health system should be amplified and further strengthened before they come under increased pressure.

The worst-case scenario imaginable right now is the entry of some more dangerous, mutated version of coronavirus into one of the very poor countries in the world with a dysfunctional health system. Such countries cannot implement satisfactory quality epidemiological surveillance measures. Then, a more dangerous version of the virus would likely infect a larger percentage of the country’s population relatively quickly. Panic would ensue, probably also a black market for the transport of migrants to other countries. Then, a more severe variant of COVID-19 would start to expand uncontrollably and in a whole new way.

In the event of any of these unfavourable developments, all of which are unfortunately possible though not likely, an entirely new protection strategy would be required. Each country will have its own approach. When the death toll in each of the affected countries begins to rise so much as to cause fear among the population, people will become increasingly willing to undertake much stricter epidemiological measures. In such a case, an increasing number of countries will resort to a solution that has proven effective in Wuhan – ie, declaring large, very strict quarantines. It is essential to buy time in such quarantines so that health systems do not become overburdened. We should then anticipate the end of winter and hope that there would be evidence of possible seasonality of this virus. It may then begin to spread in a weakened manner or disappear altogether, at least until next winter.

## 17. WITH THESE REASONS FOR CAUTION AND A NUMBER OF ADVERSE SCENARIOS, IS THERE ANY REASON FOR POSSIBLE OPTIMISM?

There are at least several reasons for optimism. First of all, epidemiological surveillance and “first lines of defence” are currently in place throughout the European Union. If they work well in most countries, it is possible that their outbreaks will be controlled and would not enter a phase of exponential growth in the number of infected cases. In the most favourable scenario, with this retention, this novel coronavirus would show seasonality. Then, it would gradually disappear from circulation among humans with changes in nature characteristic of late spring and summer [3]. However, this is the most favourable scenario, in which the final death toll would be much lower than that already caused by the flu this year.

However, if the front line of defence and epidemiological surveillance is broken through by the virus, then governments will resort to strict measures to ban assemblies and organise quarantines, as the Chinese did. Several models done in recent weeks indicate that strict quarantine should completely suppress the spread of this coronavirus within up to three months [21]. This is exactly what we have already seen in China. That is why it seems that one great positive lesson of this pandemic is that humanity today would be able to survive even more dangerous infectious diseases than COVID-19 with strict quarantine, in which people would remain until scientists developed vaccines. In this unusual situation this answer has been revealed to us all.

Finally, the tireless work of numerous scientists currently testing over a hundred drugs against this virus, as well as at least eleven experimental vaccines, should be noted [22]. It is not impossible that some existing medicines may show some effect and be repurposed to treat COVID-19. Also, vaccines should become available over time. In this unusual situation, emergence of an effective drug or a vaccine could make a sudden and important difference.

## 18. APART FROM THE APPARENT EFFECTIVENESS OF QUARANTINE IN CHINA, CAN WE DRAW ANY FURTHER LESSONS FROM THIS PANDEMIC?

We should try to find something good in all the bad things that seem to be happening. Many people may finally realise how dangerous flu is. More of them may start getting vaccinated against it. Each year, the flu kills between 250 000 and 650 000 people worldwide [18].

In China, which is one-sixth of the world’s population, the death toll from COVID-19 could be stopped below 5000 by the Wuhan quarantine. If all other countries could implement anti-epidemic measures like China, then the death toll from COVID -19 could be at most six times higher, ie, up to 30 000. That would be ten times fewer deaths than the total number of deaths caused annually by seasonal influenza. Unfortunately, many countries will not be able to follow China’s example closely and will have uncontrolled outbreaks if the warmer season does not stop the spread of the virus.

Furthermore, if the virus continues to spread throughout 2020, it will demonstrate in a very cruel way how well the leadership and public health systems of individual countries function. It will be possible to produce performance charts for each country in controlling this new infectious disease, given the population size and age structure. These will be very important lessons to learn in preparation for a future pandemic, which could be even more dangerous.

Additionally, viruses generally spread by contact. This means that it is good to be reminded that hands should be regularly and properly washed during epidemics and touching surfaces that many people touch should be avoided (knobs, handrails, ATMs). Shaking hands should also be avoided and we should keep at least two steps away from people who have symptoms of respiratory infections. It is also advisable to regularly ventilate all living quarters. It is also helpful to strengthen personal immunity with sufficient sleep, exercise and good nutrition.

## 19. ARE THERE ANY REAL SURPRISES FOR SCIENCE RELATED TO THIS PANDEMIC, AT LEAST FOR NOW?

We all hope at this point (March 7, 2020) that there would be very few. I explained why it is no surprise that after the previous six coronaviruses, the seventh has now also managed to infect humans. Nor is its spread rate a surprise, as there are both more and less infectious respiratory viruses [3]. It would be somewhat surprising for epidemiologists if the total number of infected people in the population was not significantly higher than the number of registered infected people. In such occurrence, case-fatality rate would indeed become significantly higher than that of the flu. We should continue to await the information of well-conducted studies on this key question.

Perhaps the biggest surprises so far have been related to the clinical course of COVID-19 rather than its epidemiology. For now, clinicians in China have reported that registered infected people report to the hospital rather late, on average after as many as 9-12 days of home care [23]. This may reflect their fear of being admitted to the hospital during an epidemic, but it may also be an interesting feature of an illness with slow development in comparison to other respiratory infections. Furthermore, fever does not appear to always accompany other symptoms of the disease in the first few days, making it difficult to identify possibly contagious cases by controlling people’s temperatures [23].

The media has also reported on the possible return of the virus after suffering from the infection in some cases [24]. For now, it is hard to know how common these cases are, and how many exceptions there are. It is often wise to rest well after many viral respiratory infections to allow the body to recover after the infection has ended. It is unknown whether this infection can return if the immune system has not completely removed the virus from the body. But with all new and unknown viruses such surprises are possible, so one should be careful until the pandemic is over.

## 20. WHAT ARE THE CLOSING MESSAGES AT THE END OF FEBRUARY 2020?

In all the answers provided in this editorial I endeavoured to convey the most likely scientific explanations for the abundant information that is published about COVID-19 in the domestic and international media. Over time, some of the likely scientific explanations may need to be modified in the light of new information. One should not forget that this is a new and unknown virus. Therefore, surprises and deviations from the expected scenarios are in principle possible. That is why I emphasise that caution is needed, but not panic. The community of scientists and health experts will continue to closely monitor the further development of the pandemic.

Obviously, we need to prepare for a very serious flu-like illness against which no-one will be able to be vaccinated against for quite a while. Therefore, elderly people and those with underlying illnesses should be extremely careful because the infection is the most dangerous for them. Having gained our first knowledge of COVID-19, we now need to concentrate on preventing the spread of the virus and buying time until the arrival of warmer days. Then, we might be lucky enough to see the virus slowly disappearing due to seasonality. Unfortunately, we cannot know that right now. From everything written here, it should be understood that all measures of active searching and the isolation of affected patients and their contacts are justified.

These also include bans on gathering together larger groups of people, as well as possible quarantine if the epidemic starts to elude epidemiological control. Particularly, older people should be looked after because the probability of a bad outcome increases significantly with age. In addition, those with heart disease, diabetes, or undergoing cancer treatment should take special care.
